# Potential application of ChatGPT in *Helicobacter pylori* disease relevant queries

**DOI:** 10.3389/fmed.2024.1489117

**Published:** 2024-10-10

**Authors:** Zejun Gao, Jinlin Ge, Ruoshi Xu, Xiaoyan Chen, Zhenzhai Cai

**Affiliations:** Department of Gastroenterology, Second Affiliated Hospital and Yuying Children’s Hospital of Wenzhou Medical University, Wenzhou, China

**Keywords:** *Helicobacter pylori*, intrafamilial transmission, ChatGPT, large language model, artificial intelligence

## Abstract

**Background:**

Advances in artificial intelligence are gradually transforming various fields, but its applicability among ordinary people is unknown. This study aims to explore the ability of a large language model to address *Helicobacter pylori* related questions.

**Methods:**

We created several prompts on the basis of guidelines and the clinical concerns of patients. The capacity of ChatGPT on *Helicobacter pylori* queries was evaluated by experts. Ordinary people assessed the applicability.

**Results:**

The responses to each prompt in ChatGPT-4 were good in terms of response length and repeatability. There was good agreement in each dimension (Fleiss’ kappa ranged from 0.302 to 0.690, *p* < 0.05). The accuracy, completeness, usefulness, comprehension and satisfaction scores of the experts were generally high. Rated usefulness and comprehension among ordinary people were significantly lower than expert, while medical students gave a relatively positive evaluation.

**Conclusion:**

ChatGPT-4 performs well in resolving *Helicobacter pylori* related questions. Large language models may become an excellent tool for medical students in the future, but still requires further research and validation.

## 1 Introduction

*Helicobacter pylori* (HP) is a gram-negative bacterium transmitted through the fecal–oral route that infects the human gastric mucosa epithelium and affects 50% of the world’s population, especially in developing countries, due to unhealthy dietary habits1. Long-term HP infection may lead to several gastrointestinal diseases, such as chronic inflammation, peptic ulcers, gastric cancer, and mucosa-associated lymphoid tissue lymphoma (1, 2). The World Health Organization listed it as a class I carcinogen for gastric cancer in 1994 (3). In addition to traditional test-and-treat and screen-and-treat strategies, family-based control and management has been proposed as a third approach, which is not affected by HP infection rates (4–6). However, owing to differences in education among societies, the popularity of HP-related knowledge still remains a major problem.

Recently, with the progress of technology and the rapid development of artificial intelligence (AI), enormous changes have taken place in different areas of the world, and the medical field is no exception. The application of AI in medicine is expanding in many fields, including intelligent screening, intelligent diagnosis, risk prediction and adjuvant therapy (7–9). At the same time, there has been an interest in ChatGPT in the gastroenterology community. Gravina et al. analyzed this tool showed some attractive potential in addressing IBD related issues, while having significant limitations in updating and detailing information and providing inaccurate information in some cases (10). Among them, ChatGPT, launched by OpenAI on November 30, 2022, is a new type of natural large language model (LLM) that performs well in medical education and training (11). To date, ChatGPTs have successfully passed various large medical licensing exams and other medical tests (12–15).

Therefore, LLM could be considered an interactive information resource for patients with HP infection or their families, as well as a tool for clinicians, but its ability to guide HP management is uncertain. This study was designed to assess the potential medical capacity of LLM for HP-related questions among both experts and ordinary people.

## 2 Methods

### 2.1 Study design

We formulated a series of HP-related questions on the basis of the latest relevant guidelines (6, 16, 17) and clinical experience (at least 15 years’ clinical HP work), involving life (Q1-5), test (Q6-13), and treatment guidance (Q14-22). Each question was submitted to ChatGPT-4 (18) (version 3/14/2023)^1^ three times during independent interactions without intervening feedback to assess repeatability. Nevertheless, the evaluation of model performance was planned to be restricted to the analysis of only the initial run (Answer 1). The whole process took place from April 28, 2024, to May 07, 2024. To prevent LLM from dodging medical questions, we inform ChatGPT-4 in advance that “Now that you are a professional gastroenterologist, meanwhile you have mastered the latest guidelines about *Helicobacter pylori*. Please answer the following questions.”

### 2.2 Comparative analysis of answers

A temporary assessment team, established by three HP experts, evaluated the capacity (accuracy, completeness, usefulness, comprehension, and satisfaction) of the responses, while 14 ordinary people, as nonexperts, were divided into seven medical students groups and seven nonmedical groups and evaluated for usefulness and comprehension only. All the dimensions were scored via a Likert scale (Table 1).

**Table 1:** Likert scales of every dimension.

Score	Accuracy rating by a five-point Likert scale
1	Completely incorrect
2	More incorrect than correct
3	Approximately equal correct and incorrect
4	Mostly accurate, with some slight inaccuracies or irrelevant information
5	Correct
Score	Completeness rating by a three-point Likert scale
1	Incomplete, addresses some aspects of the question, but significant parts are missing or incomplete
2	Generally complete, with the minimum amount of information
3	Very complete, addresses all aspects of the question
Score	Usefullness rating by a three-point Likert scale
1	No guidance
2	Containing only generic information or guidance
3	Containing some specific guidance
Score	Comprehension rating by a three-point Likert scale
1	Difficult to understand
2	Partly difficult to understand
3	Easy to understand
Score	Satisfaction rating by a five-point Likert scale
1	Very dissatisfied
2	Dissatisfied
3	Moderate satisfied
4	Satisfied
5	Very satisfied

### 2.3 Statistical analysis

SPSS 26.0 software (IBM Corp.) was used for statistical analysis, and GraphPad Prism 9.5 (GraphPad Software, Inc.) was used for data visualization and graph plotting. When *p* < 0.05, the difference was considered statistically significant. The Kolmogorov–Smirnov test was used to check whether the data were normally distributed, and Levene’s test was used for homogeneity of variance. The least significant difference (LSD) test and Kruskal Walls test were used for pairwise comparisons. The consistency of scores among multiple raters was evaluated by Fless’s kappa.

## 3 Results

### 3.1 Response repeatability

Table 2 and Supplementary Table 1 list each preset question and the ChatGPT-4 answer for this project. When the same question was submitted to ChatGPT-4 independently, 86.36% (19/22) of the questions received responses that were generally consistent with the previous answers. The answers to questions 3, 12 and 19 reveal subtle inconsistency in some of the details. Regarding dietary of HP patients, ChatGPT-4 focused on the healthy diet, and the third answer of Q3 mentioned pickled foods. In terms of false negatives explanation, the last answer involved the influence of bismuth-containing compounds (Q12). For people of penicillin allergic, ChatGPT had a different advice. The last answer provided the most solutions, including the high-dose dual therapy. In addition, it focused on differences in antibiotic resistance patterns (Q19).

**Table 2:** Questions imported into ChatGPT-4.

Number	Questions
Q1	Today I had a medical check-up at my company, and the doctor said I have HP^1^. What is HP?
Q2	What impact will HP have on my life, studies, or work?
Q3	I’ve been diagnosed with HP, are there any dietary considerations I need to be aware of?
Q4	Can HP cause cancer?
Q5	I tested positive for HP, is it hereditary?
Q6	I tested positive for HP, should my family members also get tested?
Q7	I recently have acid reflux and have been taking omeprazole, should I get tested for HP?
Q8	I’ve recently had bad breath, do I need to get tested for HP?
Q9	I had a cold last week and took cold medicine, can I still get tested for HP?
Q10	Does our whole family need to get tested for HP and treated together?
Q11	I need to get tested for HP, what tests can I do? What are the advantages and disadvantages of these tests?
Q12	My C13 breath test result was negative, does this mean I do not have HP?
Q13	My C13 breath test value is very high, does that mean it is severe?
Q14	My C13 breath test result is positive, can I avoid treatment? If not, please provide a specific treatment plan.
Q15	My blood test results show HP antibody positive, do I need treatment?
Q16	An elderly family member tested positive for HP last week, do they need treatment?
Q17	My child tested positive for HP last week, do they need treatment?
Q18	My wife is pregnant and tested positive for HP last week, does she need treatment?
Q19	I tested positive for HP, but I’m allergic to penicillin, what should I do?
Q20	I took the prescribed antibiotics, does this mean I’m cured?
Q21	I followed the treatment and took antibiotics, why is the follow-up test still positive? What should I do next?
Q22	I heard that potassium-competitive acid blockers is a good drug. Can this be used to treat HP infection?

1 HP, *Helicobacter pylori*.

### 3.2 Response length analysis of ChatGPT-4

Table 3 presents the response lengths of ChatGPT-4 across each response. The average word count was 195.94 ± 52.96. Among each response, the average word count and SD of answers 1 to 3 were 198.91 ± 58.51, 190.82 ± 52.25, and 198.09 ± 69.95, respectively (*p* > 0.05). The average character count was 1302.30 ± 382.56. Among each response, the character average count and SD of answers 1 to 3 were 1314.09 ± 407.04, 1278.73 ± 376.14, and 1314.09 ± 493.36, respectively (*p* > 0.05).

**Table 3:** Length analysis of ChatGPT-4 answers.

Response length of GPT-4	Word count (SD)	Minimum	Maximum	Character count (SD)	Minimum	Maximum
Answer1	198.91 (58.51)	80	312	1314.09 (407.04)	541	2032
Answer2	190.82 (52.25)	98	303	1278.73 (376.14)	642	2030
Answer3	198.09 (69.95)	107	363	1314.09 (493.36)	716	2,374
Total	195.94 (52.96)	95	286	1302.30 (382.56)	633	2045.67
*p* value	0.969			0.991		

### 3.3 Interrater reliability

Fleiss’ kappa was used to assess the consistency of the ratings. The Fleiss’ kappa evaluations for “accuracy (Fleiss’ kappa: 0.690, *p* < 0.001)” was rated as “substantial agreement,” while “completeness (Fleiss’ kappa: 0.456, *p* < 0.001),” “usefulness (Fleiss’ kappa: 0.564, *p* < 0.001)” and “satisfaction (Fleiss’ kappa: 0.580, *p* < 0.001)” were rated as “moderate agreement,” and “comprehension (Fleiss’ kappa: 0.302, *p* = 0.014)” was rated as “fair agreement” (Supplementary Table 2).

### 3.4 Evaluation of the ChatGPT-4 responses in each dimension by the experts

**Figure 1:**
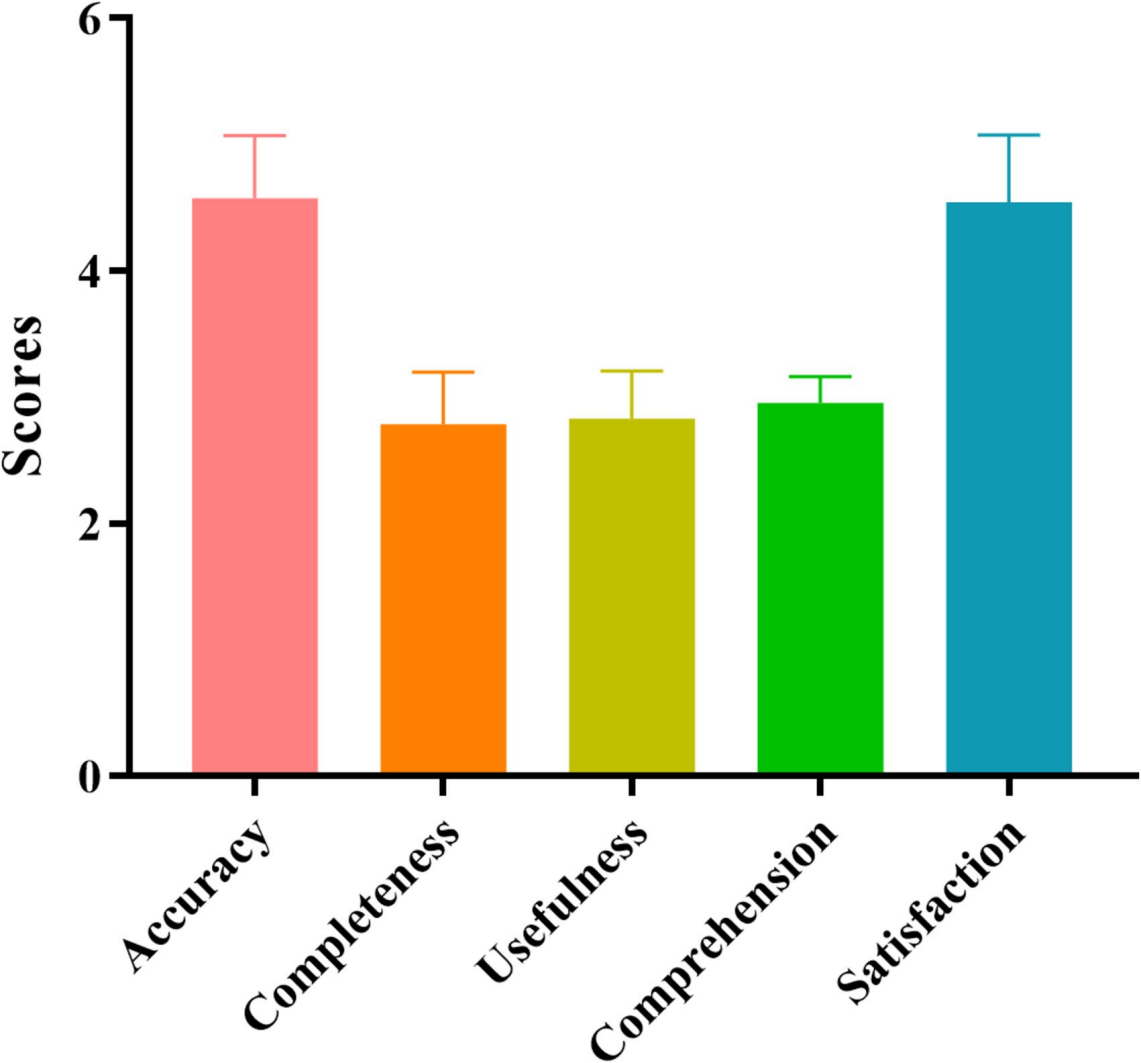
Different dimension analysis of ChatGPT-4 answers to HP queries by experts.

As shown in Figure 1 and Table 4, the average quality scores of accuracy, completeness, usefulness, comprehension, and satisfaction for ChatGPT-4 were 4.58 ± 0.50, 2.79 ± 0.41, 2.83 ± 0.38, 2.95 ± 0.21, and 4.55 ± 0.53, respectively. The score for life guidance was higher than that for test and treat guidance, but the differences were not significant (*p* > 0.05).

**Table 4:** Each dimension scores analysis of ChatGPT-4 answers by experts (Average ± SD).

Dimension	Overall	Life guidance	Test guidance	Treatment guidance	*p* value
Accuracy	4.58 ± 0.50	4.73 ± 0.46	4.50 ± 0.51	4.56 ± 0.51	0.548
Completeness	2.79 ± 0.41	2.80 ± 0.41	2.79 ± 0.41	2.78 ± 0.42	0.999
Usefulness	2.83 ± 0.38	2.93 ± 0.26	2.79 ± 0.41	2.81 ± 0.40	0.697
Comprehension	2.95 ± 0.21	3.00 ± 0.00	3.00 ± 0.00	2.89 ± 0.32	0.212
Satisfaction	4.55 ± 0.53	4.73 ± 0.46	4.46 ± 0.51	4.52 ± 0.58	0.434

### 3.5 Performance of ChatGPT-4 among ordinary people

**Figure 2:**
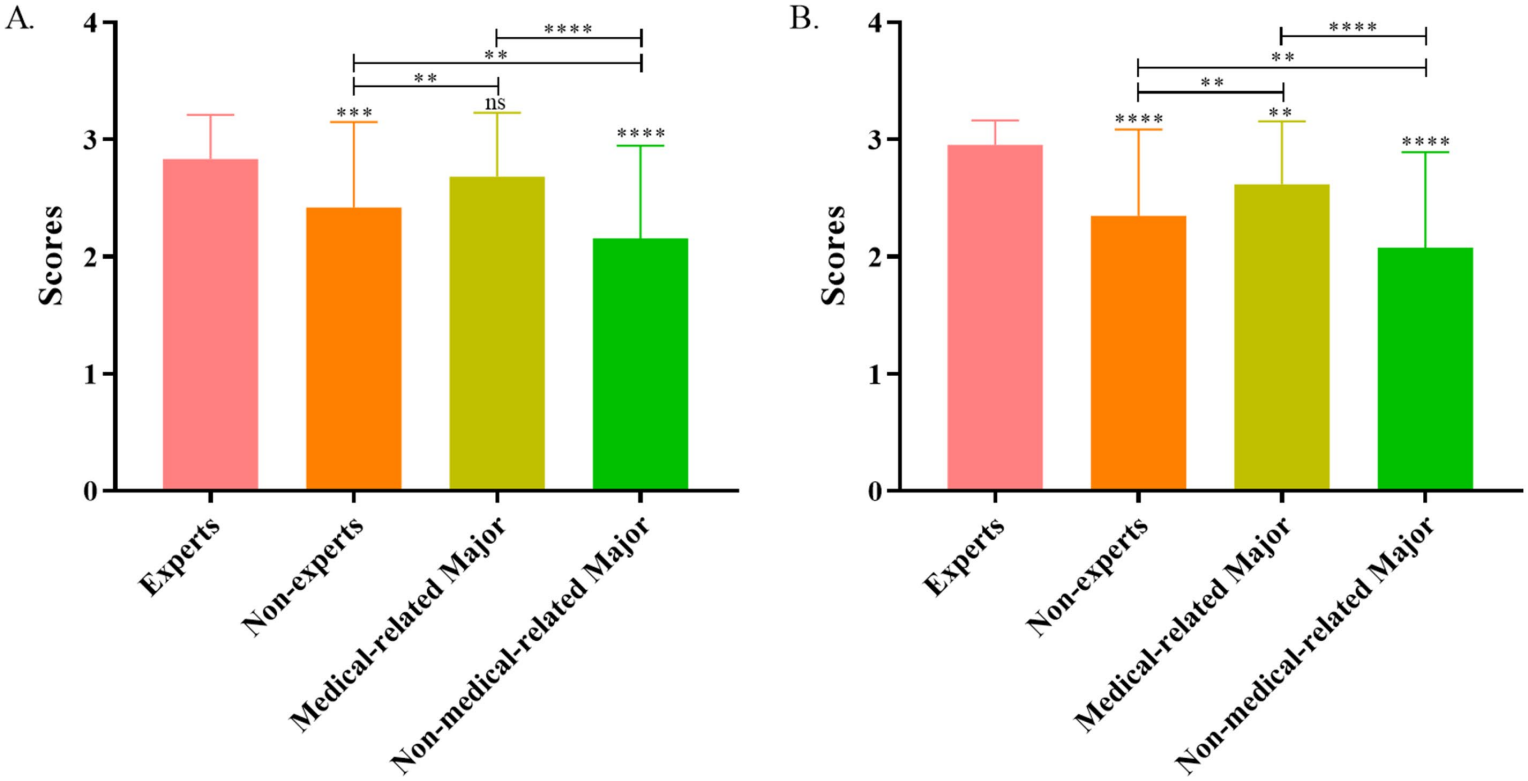
Usefulness (A) and comprehension (B) score analysis of ChatGPT-4 answers between experts and nonexperts.

The average overall usefulness score for the ChatGPT-4 by experts was 2.83 ± 0.38, which was significantly higher than that of nonexperts (2.42 ± 0.73; *p* < 0.001). The scores of medical students (2.68 ± 0.54) were higher than nonmedical people (2.16 ± 0.79; *p* < 0.001; Figure 2A).

In terms of comprehension scores, the average overall score for experts was 2.95 ± 0.21, which was significantly higher than that for nonexperts (2.35 ± 0.74; *p* < 0.001). Further analysis revealed that medical majors scored higher than nonmedical majors did (*p* < 0.01; Figure 2B).

## 4 Discussion

Although the infection rate of HP in China has been slowly declining over the past three to four decades, it is still a major health threat to families and society in China (19). These HP infected people develop different types and degrees of gastrointestinal and extragastrointestinal diseases, such as dyspepsia, chronic gastritis, peptic ulcers, gastric cancer, iron deficiency anemia, and idiopathic thrombocytopenic purpura (16, 20). However, the public’s understanding of HP is not enough. Previous studies have demonstrated the promising prospects of ChatGPT in medicine, and some have assessed the ability of ChatGPT-3.5 to address HP-related queries (21–23). However, previous studies have evaluated only the accuracy and repeatability of the ChatGPT-3.5 model, and the set of questions has neglected the family cluster characteristics associated with HP infection (23). Therefore, the purpose of this project is to determine whether chaGPT-4 can solve HP-related questions and explore the potential applications of LLM among ordinary people.

As an important tool, the LLM of AI is gradually affecting every field among human beings. According to the HP guidelines and clinical experience, we designed several HP-related issues. We found that ChatGPT-4 performed well in terms of the repeatability of each prompt. However, there are still some subtle differences that need to be carefully identified, which is obviously better than the previous research results of ChatGPT-3.5 (24). These findings suggest that LLMs, such as ChatGPT-4, have significant potential medical applications in the future.

We found that the ChatGPT-4 resulted in high scores on accuracy, completeness, usefulness, comprehension and satisfaction dimensions in terms of performance on HP-related questions. After the questions were classified by life, test and treatment guidance, the score for life guidance was highest, but the differences were not significant. This illustrates that the ability of ChatGPT-4 to respond to HP-related issues is good and that ChatGPT-4 has a good breadth of knowledge. Owing to its vast dataset and continuous learning ability, ChatGPT-4 performs well in processing medical information. In addition, the integration of an advanced reasoning mechanism and strict adherence to the guidelines enabled ChatGPT-4 to address complex clinical demands. Importantly, the inclusion of a substantial volume of up-to-date medical training data and the assimilation of lessons from practical application experiences collectively have improved the quality and relevance of the responses provided by ChatGPT-4 (25, 26). ChatGPT would firstly respond according to guidelines. But there is a time limit for model training. At the same time, ChatGPT would take patient-specific factors into consideration. When mentioned the heredity (Q5), it supplemented the transmission route of HP after it denied the heredity of HP. Involved in HP treatment (Q13), it provided alternatives for penicillin allergy patients, while we did not ask about how to resolve allergy patients beforehand. Finally, ChatGPT will lead you to follow doctor’s advice. In addition, multiple responses mentioned relevant guidelines and these contents of the repeated responses were roughly the same. ChatGPT-4 did not clearly state which literature was cited, needing a step further prompt. This means that LLMs, such as ChatGPT-4, can become excellent tools for both doctors and patients, showing considerable development prospects. However, these outputs by ChatGPT were needed suspicion, although it was rated well by the experts of our research.

We further collected and analyzed the masses to assess the comprehensiveness and usefulness of the data. The analysis revealed that the usefulness and comprehension results of ChatGPT-4’s replies on HP-related queries among nonexperts were not as good as those among experts. Some individuals thought that these answers were too obscure and lacked significance. This gap widened when nonexperts were divided into medical professions and nonmedical majors. In terms of usefulness, although the scores of medical-related majors were lower than those of expert majors, the difference was not statistically significant. In contrast, perhaps owing to the inherent difficulty and threshold of medical knowledge, the average scores were moderate among nonmedical majors, with scores of only 2.08 for comprehension and 2.16 for usefulness. In fact, these results are very easy to understand. HP experts have mastered the latest advances in HP research and have pivotal positions in this field. Compared with ordinary people, those who have medical knowledge, who have a certain knowledge base, can more easily understand the answers. However, the public, especially those in developing countries, generally lack medical knowledge, and many people are still illiterate. The resolution of their problems is of utmost importance, as they constitute the main body of the world. Despite the presence of a hierarchical diagnosis model in China, it still cannot change the phenomenon whereby large hospitals in cities are full of patients and small hospitals are empty. This not only imputes the scarcity and uneven distribution of medical resources but also contributes to the imperfect knowledge system of doctors in small hospitals (27). As the birth of LLM, these obsessions may be gradually resolved, which will help the knowledge acquisition of the masses and the rapid progress of medical beginners. On the other hand, LLM may be able to shorten the distance between doctors and patients and increase medical efficiency. Therefore, regardless of whether LLM replaces clinicians, it is likely to become an important consultation option for ordinary patients in the future.

Focusing on the familial aggregation of HP infection, we asked the corresponding questions. Considering questions 6 and 10, ChatGPT-4 suggested that family members of HP patients should only be tested and treated unless they have symptoms or a family history of gastric cancer. However, this reply was too narrow. Mounting evidence has demonstrated that the main route of transmission of HP is through the mouth and that HP infection is associated with a family cluster (28, 29). In addition to traditional test-and-treat and screen-and-treat strategies, a third new family-based strategy has recently been proposed (6). The new strategy targets HP-infected individuals within the family, and its scope of application is not affected by HP infection rates. With respect to historical traditional differences, some families in China usually share foods in the same dish or bowl, sometimes using the same utensils, which are sources of HP cross-contamination. Thus, the need for family-based test and treatment becomes the key. The “Chinese Consensus Report on Family-Based *Helicobacter pylori* Infection Control and Management (2021 Edition)” suggests that, unless there are competing considerations, family-based HP infection management and the eradication of HP infection are recommended, which is helpful for reducing the chance of transmission of infection and reinfection after its eradication (6). Compared with the consensus, the answers of ChatGPT-4, which ignore cultural diversity and skip the new strategy, seem to be imperfect. Therefore, excessive care must be taken when AI models are employed in practical medical fields to ensure that inaccurate information is not generated due to model limitations.

Moreover, in terms of treatment regimen, we focused on potassium-competitive acid blockers (P-CABs) in HP treatment (Q22). As a new regimen, the research volume of relevant P-CAB-based HP therapy were not large. Kanu et al. found P-CAB-based therapy had a promising effect on HP eradication (30). Distinguishing itself from conventional proton pump inhibitors, this class of drugs can have a more durable and stable acid control effect. P-CAB exhibits versatile clinical applications, encompassing the treatment of gastroesophageal reflux disease, peptic ulcer disease, and HP eradication therapy (31, 32). For P-CAB aspect, ChatGPT-4’s answers showed a good performances (Q22). However, ChatGPT-4 did not mention this treatment alternate among other treatment-related questions (Q14-21). ChatGPT will choose those widely recognized treatments, such as triple therapy, rather than those under investigation. There was no real-time access to the internet when responding to queries, so its knowledge base is fundamentally limited (33). That is, ChatGPT may not derive enough accurate information until updates has been fed to the model (34). This also becomes a constraint, which may not be able to keep up with the big explosion of information.

In addition, as a powerful peer-to-peer fast feedback interactive program, LLM can help people quickly acquire needed information, speed up life and work efficiency. However, LLMs may be addictive as drugs, causing the public to gradually lose their ability to think by themselves. Not long ago, owing to the sudden collapse of the ChatGPT website, people expressed on social media that their lives and jobs were unable to operate completely anymore (35). Therefore, regardless of how AI affects our lives in the future, it is crucial to keep a clear mind to judge the progress of science.

This task still has several limitations. This article simply probes the potential medical applications of LLM in the future through the replies of ChatGPT-4 to HP-related questions. This study did not examine other AI models, nor did it examine responses to other clinical questions.

## 5 Conclusion

ChatGPT-4 performs well in resolving HP related questions, which is expected to be a convenient and effective tool for people.

## Data Availability

The original contributions presented in the study are included in the article/Supplementary material, further inquiries can be directed to the corresponding authors.

## Footnotes

^1^https://openai.com/gpt-4
